# Permafrost thaw driven changes in hydrology and vegetation cover increase trace gas emissions and climate forcing in Stordalen Mire from 1970 to 2014

**DOI:** 10.1098/rsta.2021.0022

**Published:** 2022-01-24

**Authors:** Ruth K. Varner, Patrick M. Crill, Steve Frolking, Carmody K. McCalley, Sophia A. Burke, Jeffrey P. Chanton, M. Elizabeth Holmes, Isogenie Project Coordinators, Scott Saleska, Michael W. Palace

**Affiliations:** ^1^ Department of Earth Sciences, Oceans and Space, University of New Hampshire, Durham, NH 03824, USA; ^2^ Institute for the Study of Earth, Oceans and Space, University of New Hampshire, Durham, NH 03824, USA; ^3^ Department of Physical Geography, Stockholm University, Stockholm, Sweden; ^4^ Department of Geological Sciences, Stockholm University, Stockholm, Sweden; ^5^ Bolin Centre for Climate Research, Stockholm University, Stockholm, Sweden; ^6^ Thomas H. Gosnell School of Life Sciences, Rochester Institute of Technology, Rochester, NY 14623, USA; ^7^ Department of Earth, Ocean and Atmospheric Science, Florida State University, Tallahassee, FL 32306-4350, USA; ^8^ Division of Science and Math, Tallahassee Community College, 444 Appleyard Drive, Tallahassee, FL 32304, USA; ^9^ Department of Ecology and Evolutionary Biology, University of Arizona, Tucson, AZ 85721, USA; ^10^ Department of Microbiology, The Ohio State University, Columbus, OH 43210, USA; ^11^ Centre for Microbiome Research, School of Biomedical Science, Translational Research Institute, Queensland University of Technology, Woolloongabba, QLD 4102, Australia

**Keywords:** methane, radiative forcing, Arctic, permafrost, remote sensing, landcover

## Abstract

Permafrost thaw increases active layer thickness, changes landscape hydrology and influences vegetation species composition. These changes alter belowground microbial and geochemical processes, affecting production, consumption and net emission rates of climate forcing trace gases. Net carbon dioxide (CO_2_) and methane (CH_4_) fluxes determine the radiative forcing contribution from these climate-sensitive ecosystems. Permafrost peatlands may be a mosaic of dry frozen hummocks, semi-thawed or perched sphagnum dominated areas, wet permafrost-free sedge dominated sites and open water ponds. We revisited estimates of climate forcing made for 1970 and 2000 for Stordalen Mire in northern Sweden and found the trend of increasing forcing continued into 2014. The Mire continued to transition from dry permafrost to sedge and open water areas, increasing by 100% and 35%, respectively, over the 45-year period, causing the net radiative forcing of Stordalen Mire to shift from negative to positive. This trend is driven by transitioning vegetation community composition, improved estimates of annual CO_2_ and CH_4_ exchange and a 22% increase in the IPCC's 100-year global warming potential (GWP_100) value for CH_4_. These results indicate that discontinuous permafrost ecosystems, while still remaining a net overall sink of C, can become a positive feedback to climate change on decadal timescales.

This article is part of a discussion meeting issue ‘Rising methane: is warming feeding warming? (part 2)’.

## 1. Introduction

Accelerated climate warming in the Arctic has led to permafrost thaw which results in a deeper active layer, changes in soil moisture and hydrology and subsequent vegetation community shifts [1–3]. These changes alter the rates of production, consumption and net emission of radiatively important trace gases like carbon dioxide (CO_2_) and methane (CH_4_): both gases play important roles in the radiative balance and atmospheric warming. The net uptake or release of these gases from thawing ecosystems determine the feedback to climatological response of these vulnerable landscapes [4–6]. Quantifying landcover transition during thaw is essential to determining the balance of trace gas emissions of these climate-sensitive landscapes [7,8].

Permafrost peatlands are a mosaic of frozen hummocks or palsas, semi-thawed sphagnum dominated areas or bogs, fully thawed sedge dominated areas or fens and often small open water ponds formed through the collapse of permafrost [1,9]. Monitoring changes in these sub-habitats is critical to partitioning and quantifying the climate forcing fluxes from this region. Landcover classification has traditionally been determined using plot-based species cover observations and identifying plant functional groups. This requires multiple plot locations across a landscape to determine dominant landcover types. To aid in the scaling of plot-based measurements, a growing number of studies use images collected from manned and unmanned aerial systems (UASs) that provide information on the scale of centimetres [10–12] and at lower resolution, satellite remote sensing (less than 30 m) [13,14].

To determine the net exchange of CO_2_ or CH_4_ from different landcover types, researchers use eddy covariance techniques which result in integrated landscape-scale fluxes. Chamber measurements, automated or manual, can help to partition these fluxes to plant community types at metre square scales [15–17]. For open water surfaces too small for eddy covariance techniques, like those often found in thawing environments, estimates of the rate of hydrodynamic advection of trace gases are measured directly using floating chambers [18,19] or by estimating fluxes using dissolved concentration measurements in combination with transfer coefficients [20,21]. Ebullition or bubbling, another potentially dominant transport pathway of CH_4_ emission from ponds, is measured using bubble traps distributed across the water surface [22,23].

Estimating the climate forcing from trace gas emissions is an important component of the total radiative forcing of an ecosystem. Net radiative forcing can be estimated using landcover classification along with gas exchange rates and global warming potential values derived using laboratory and modelling techniques [24]. Long-term evaluation of climate forcing over the Holocene indicates that peatland formation has had a cooling impact on our climate due to the high rates of CO_2_ fixation overwhelming the warming impact of CH_4_ emissions [25,26]. Using this approach is critical for understanding the climate feedbacks of thawing permafrost regions which over time have the potential to exchange large amounts of both CO_2_ and CH_4_ with the atmosphere and with current rates of thawing shift these ecosystems from net cooling to net warming [27].

High latitude landscapes that store large quantities of carbon are experiencing change due to permafrost thaw. Quantifying changes in landcover in addition to applying accurate estimates of trace gas exchange across landcover classes is the only way to predict the impact of the changing Arctic temperatures on our future climate. Here, we present an updated vegetation composition dataset and an improved estimate of annual CO_2_ and CH_4_ exchange rates to present a time series of trace gas radiative forcing for the Stordalen Mire, a permafrost peatland located in the discontinuous permafrost zone of Northern Sweden.

## 2. Methods

### (a) Site location

**Figure 1.**
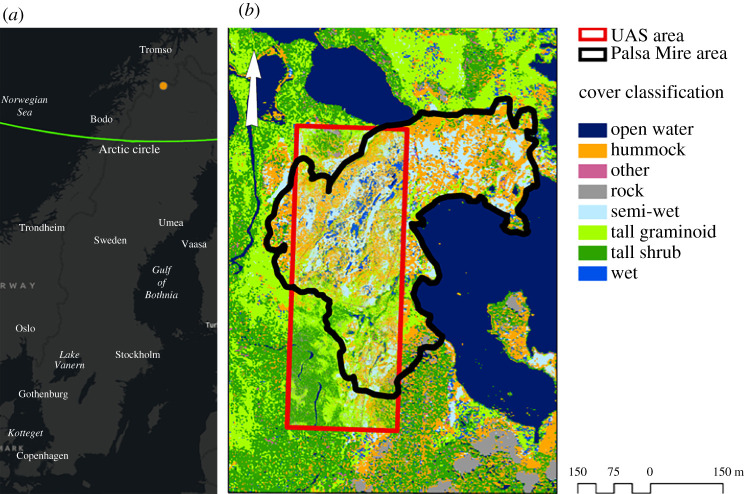
(*a*) Our study site was Stordalen Mire located in northern Sweden. (*b*) Vegetation map overlayed on a WV-2 satellite image from 2014 [11]. The rectangle represents the unpersoned aerial system (UAS) training area for the 2014 vegetation analysis. The training area was then applied to the Palsa Mire area (outlined polygon) which represents the area from the Christensen *et al.* [1] and Malmer *et al.* [28] comparison datasets. (Online version in colour.)

Stordalen Mire (68°21′ N 18°49′ E) is located in northernmost Sweden at the edge of the discontinuous permafrost zone (figure 1). Research at this site has been ongoing since the early 1900s and monitoring efforts include measurement of meteorological parameters as well as carbon and radiative balance specifically over the last half century [29]. Permafrost in the region has been thawing in the past few decades [30]. This permafrost peatland is located at the edge of Lake Torneträsk and is surrounded by small, shallow post-glacial lakes. The peatland is composed of elevated drained palsa areas underlain by ice-rich permafrost, ombrotrophic wet sphagnum dominated areas (bogs), permafrost-free sedge dominated sites (fens) and open water ponds both persistent and formed through permafrost thaw. Landcover classification at Stordalen Mire was determined using plot-based techniques [31] and vegetation maps were constructed using these classifications along with plot-based data and aerial photos for 1970 and 2000 [1,32,33] (electronic supplementary material, table S1). Radiative forcing estimates using these landcover classifications and autochamber and eddy covariance trace gas fluxes were previously reported for 1970 and 2000 [32].

### (b) Vegetation species composition

Using an artificial neural network (ANN) and UAS imagery collected at Stordalen Mire in 2014, a previous study developed a landcover map focused on vegetation cover types representing permafrost thaw stages [11]. As part of that study, vegetation plots were geolocated and landcover classes were recorded. We developed a new ANN that used these field plots and high-resolution optical satellite imagery (WorldView-2 (WV-2)) to expand the domain of the vegetation cover type to the larger Stordalen Mire area. WV-2 imagery from 8 August 2014 was orthorectified and used in our expanded analysis. All eight spectral bands available in the WV-2 imagery (spatial resolution 2 m) were used in the ANN. Our ANN approach was similar to that used by Palace *et al*. [11]. We split the data into model training and validation datasets using a method called k-fold holdback, which splits the data into a specific number of groups which in our case was five. Our ANN used five nodes that used tanH to connect input data to classified output. We ran the ANN with 100 tours. An overfitting parameter was also used to make sure the model was flexible when applied outside the training dataset. The ANN has a generalized *r*^2^ value of 0.98 and a misclassification rate of 0.4%. We focused on the area in the polygon identified in Christensen *et al*. [1] (outlined in black; figure 1) as the permafrost palsa area at Stordalen Mire. We did this so a direct comparison with climate forcing in the Johansson *et al*. [32] paper could be conducted. We digitized the polygon from the Johansson *et al*. [32] paper and georeferenced it to our new ANN classification. We used the same classification as in Palace *et al*. [11] which was based on Sonesson & Kvillner [31] and then used by Malmer *et al*. [28]. Non-vegetation cover types (rock and man-made surfaces) were included in the classification. The classification was reduced to Open Water, Palsa (tall shrub and hummock), Bog (semi-wet and wet) and Fen (tall graminoid) to allow for comparison with previously published cover types sampled for trace gas emissions.

### (c) Radiative forcing calculations

To determine the trace gas radiative forcing for Stordalen Mire, we compiled an updated trace gas flux dataset representing major landcover classes present at the site (table 1). The trace gas exchange rates for terrestrial sites (palsa, bog and fen) are from annual estimates derived from autochamber measurements [34]. The open water CH_4_ emissions were from reported ebullitive measurements using bubble traps [22] and floating chambers [35]. Open water emissions for CO_2_ were estimated from dissolved CO_2_ measurements [36] (*p*CO_2_). Average estimates of annual trace gas exchange for both CH_4_ and CO_2_ were then applied to the local landcover area for all categories for 1970, 2000 and 2014 (electronic supplementary material, table S1). We also calculated the net ecosystem carbon balance (NECB) for each landcover type and for the mire area as a whole.

**Table 1.** Compilation of fluxes and methods for previous and current climate forcing estimates for Stordalen Mire, Sweden.

		flux rates and measurement methods^e^				
landcover class	trace gas site type	CH_4_ (gC m^−2^ d^−1^)	s.d. (gC m^−2 ^d^−1^)	CO_2_ (gC m^−2^ d^−1^)	s.d. (gC m^−2 ^d^−1^)	methods
hummock	palsa	0.002	0.0013	−0.083	0.0523	CO_2_ and CH_4_ autochambers^a^
tall shrub	palsa	0.002	0.0013	−0.083	0.0523	
wet graminoid	fen	0.13	0.0242	−0.72	0.2797	
semi-wet	bog	0.026	0.0085	−0.35	0.1039	
wet	bog	0.026	0.0085	−0.35	0.1039	
open water	pond	0.10	0.0028	0.93	0.1270	CH_4_: ebullition traps^b^, floating chambers^c^
						CO_2_: *p*CO_2_ from water samples^d^

^a^Holmes *et al*. [34].

^b^Burke *et al*. [22].

^c^Kuhn *et al*. [35].

^d^Jansen *et al*. [36].

^e^Calculated daily rates using published annual emission rates except for open water gas emission for *p*CO_2_ [36] and CH_4_ ebullition [22].

To determine the trace gas radiative forcing for Stordalen Mire, we used the 100-year GWP value for CH_4_ of 28 (without CH_4_ carbon cycle feedbacks) from the IPCC AR5 [24]. First taking annual CH_4_ flux as Mg CH_4_ m^−2^ (Mg = megagrams) then multiplying it by 28 to give Mg CO_2-equiv_ m^−2^ equivalents from CH_4_ emissions. To determine the net radiative forcing for the Mire, the CO_2_ equivalents from CH_4_ were added to the CO_2_ equivalents from CO_2_ emission estimates.

We applied rates of annual emissions as CO_2-equiv_ to landcover maps for Stordalen Mire (black outlined area in figure 1) for 1970, 2000 and 2014 for CH_4_, CO_2_ and the net radiative forcing. To develop the 1970 and 2000 maps of net emissions and radiative forcing, we digitized landcover maps from Malmer *et al*. [28]. These were cropped and georeferenced using 10 points and a nearest neighbour, with a polynomial 1 setting in QGIS [37]. We then imported these images into Google Earth Engine. Using the legend in the original image from Malmer *et al*. [28] for training data for each cover class and an additional background class, we classified the image using a support vector machine algorithm. Images were then exported and imported into QGIS, where cover types were reclassified based on emission rates. Contributions to radiative forcing from nitrous oxide (N_2_O) were not considered in this analysis in part because there are no published data that suggest emissions of N_2_O occur at Stordalen Mire and we expect that N_2_O fluxes from this landscape are low due to reduced conditions [38].

## 3. Results

### (a) Change in landcover area at Stordalen Mire

**Figure 2.**
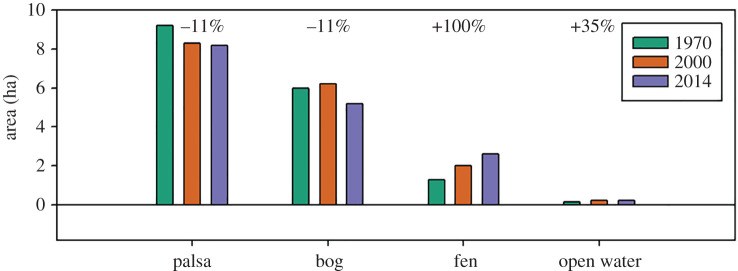
Landcover area (ha) for the Stordalen Mire (black outlined area in figure 1) for 1970, 2000 and 2014 (this study). Landcover specific areas for 1970 and 2000 are from Christensen *et al*. [1]. Numbers indicate the overall per cent change of each landcover class between 1970 and 2014 with negative values indicating a loss in landcover area and positive values indicating an increase in landcover area. (Online version in colour.)

Area estimates for major landcover classifications for 2014 compared to those reported for 1970 and 2000 [1,32] indicate that at Stordalen Mire inundated area has increased and permafrost area decreased over the past 45 years (figure 2 and electronic supplementary material, table S1). Specifically, permafrost Palsa area has decreased by 11% (a loss of 1.0 ha), while sedge dominated Fens and Open Water ponds have increased by 100% (an increase of 1.3 ha) and 35% (an increase of 0.06 ha), respectively. The rate of change was not consistent across landcover categories (electronic supplementary material, table S1), with nearly linear decreases in Palsa area and increases in Fen and Open Water area over time (figure 2; electronic supplementary material, table S1) compared to an initial increase (1970–2000) followed by a much larger decrease (1970–2000) in Bog cover. The rate of landcover change was similar for Palsa as was gained for Fen, with a 0.02 ha yr^−1^ decrease and 0.03 ha yr^−1^ increase, respectively.

### (b) Trace gas emissions and radiative forcing of Stordalen Mire

**Figure 3.**
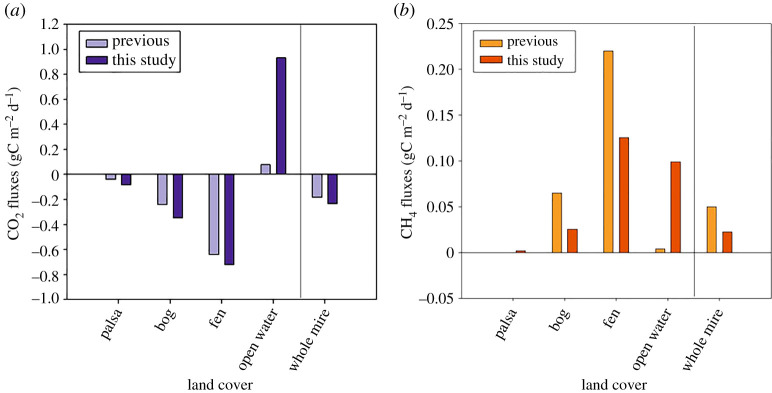
Fluxes of carbon by landcover types for (*a*) CO_2_ (gC m^−2^ d^−1^) and (*b*) CH_4_ (gC m^−2^ d^−1^) at Stordalen Mire. Positive values represent net emission from the land surface and negative values represent net uptake by the land surface. Previous study fluxes are from Christensen *et al*. [1] and Johansson *et al*. [32]. See electronic supplementary material, table S2 for values. (Online version in colour.)

Our updated CH_4_ and CO_2_ emission inventory uses annually estimated exchange rates, unlike emissions used in the previous climate forcing estimates for Stordalen Mire, which used only growing season values for trace gas exchange (table 1 and figure 3). When compared to growing season estimates, the annual numbers reported as daily rates (in gC m^−2^ d^−1^) are lower for CH_4_ emissions from both Fen and Bog landcover classes, likely because non-growing season emissions are lower due to small production rates in the ice-covered season. In all other cases (for CO_2_ and for CH_4_ from Palsa and Open Water landcover classes) the emission rates we used to estimate annual C exchange and GWP_100 are higher than those used in Johansson *et al*. [32] and Ramaswamy *et al*. [39]. Our data represent measurements that were taken in the Stordalen Mire area and use improved measurement techniques applied over the full year or during the full ice-free season for open water areas [22,35,36] (table 1). At Palsa sites, CH_4_ emissions turned from a small negative sink to a slight positive source based on autochamber measurements. Conversely, the Palsa area measurements indicate that they are a larger sink of C with a higher annual uptake rate of CO_2_.

**Figure 4.**
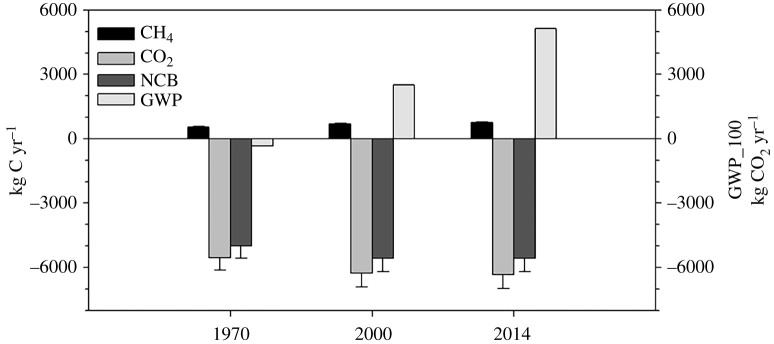
Annual CH_4_ and CO_2_ exchange and net ecosystem carbon balance (kg C yr^−1^) and radiative forcing (GWP_100 in kg CO_2-equiv_ yr^−1^) of Stordalen Mire, Sweden for 1970, 2000 and 2014.

Calculation of the annual exchange of CH_4_, CO_2_ and the neNECB for Stordalen Mire indicates that the mire remained a net sink of C showing very little variability over the 1970–2014 time period (figure 4). Mire-wide CH_4_ emissions, however, increased steadily over the 45-year period from 537 to 761 kg C yr^−1^. Carbon dioxide exchange showed an increase from 1970 to 2000 and again in 2014 although the increase was smaller than that of the previous time frame (−5600, −6300 and −6300 kg C yr^−1^). The NECB has a similar pattern to the CO_2_, varying between 5000, 5600 and 5600 kg C yr^−1^ from 1970, 2000 and 2014, respectively.

While the mire continues to be a net C sink of almost 5600 kg C yr^−1^ over the 45-year period, the net radiative forcing for Stordalen Mire has gone from slightly negative (−330 kg CO_2-equiv_ yr^−1^) to highly positive in 2000 (+2500 kg CO_2-equiv_ yr^−1^) and increased again in 2014 (5100 kg CO_2-equiv_ yr^−1^) (figure 4). The rate of increase in the GWP_100 was 0.09 kgCO_2-equiv_ yr^−1^ yr^−1^ from 1970 to 2000 and has doubled to 0.18 kg CO_2-equiv_ yr^−1^ yr^−1^ from 2000 to 2014.

**Figure 5.**
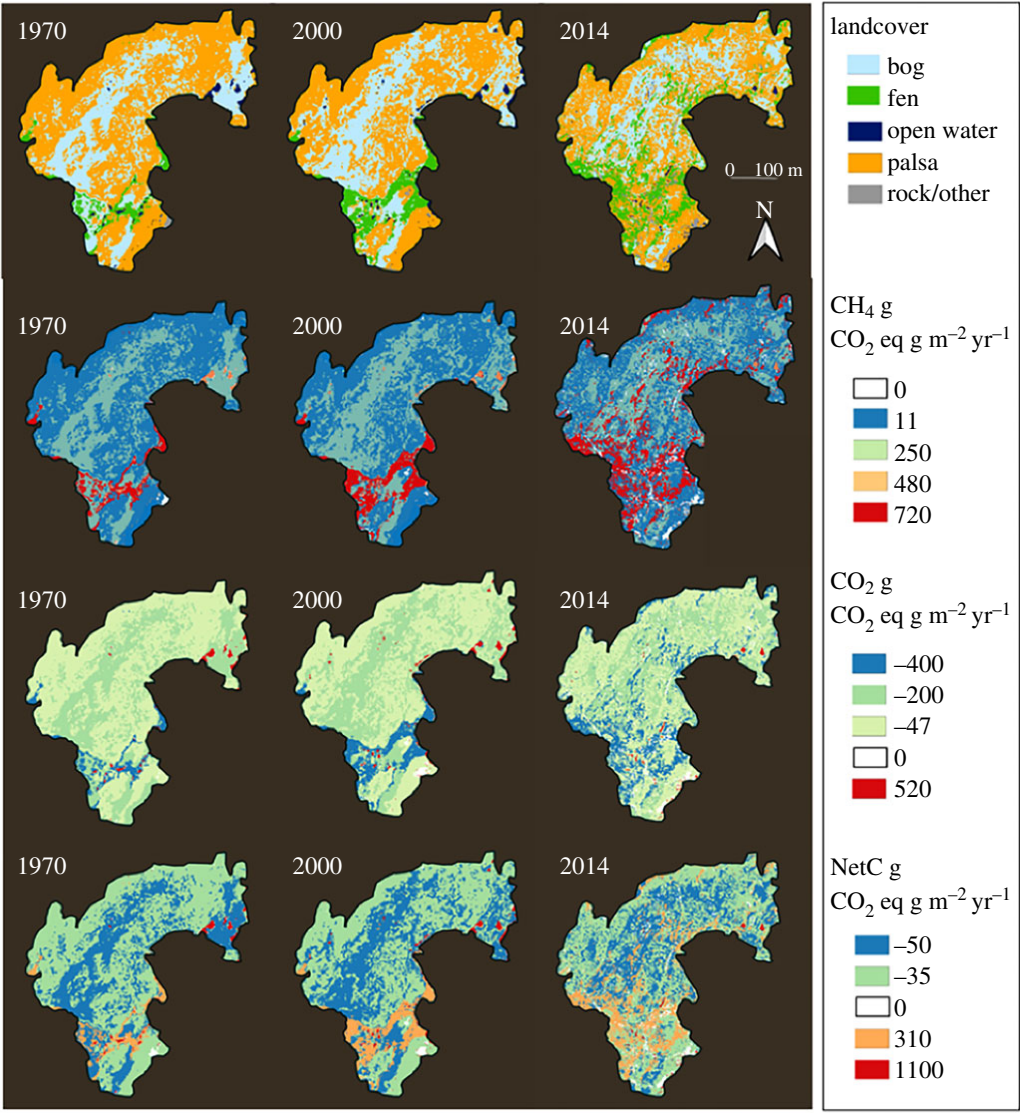
Time series of landcover, CH_4_, CO_2_ and net radiative forcing represented as g CO_2-equiv _m^−2 ^yr^−1^ at Stordalen Mire, a permafrost peatland in northern Sweden for 1970, 2000 and 2014. See black outline in figure 1 for location. (Online version in colour.)

Mapping emissions and net radiative forcing for the mire delineated area over the 1970, 2000 and 2014 time periods reveals a spatial pattern in landcover change not discernible when looking at the bulk change in landcover classes alone (figure 5). Landcover change has occurred most dramatically along the south-central portion of the mire delineated region showing the increases in fen dominated landcover which based on emissions estimates also shows increased CH_4_ emissions and CO_2_ uptake and higher net radiative forcing in this region of the mire.

## 4. Discussion

### (a) Stordalen Mire is getting wetter

As warming accelerates in the Arctic, permafrost peatlands, especially those located in the discontinuous and sporadic permafrost zones, are thawing, resulting in increasing active layer thickness and soil moisture. This thaw in turn leads to transitions in plant community composition from low productivity short stature shrubs and lichens that require drier, colder environments to more productive water tolerant mosses and sedge dominated ecosystems [3,32]. Our analysis of landcover change at Stordalen Mire from 1970 to 2014 indicates that this region is continuing the trend of transitioning from dry, permafrost dominated Palsa areas to wetter, more sedge dominated fens at rates which we can observe on decadal time scales, consistent with previous trends observed at this site and over similar time frames across the Arctic [1,3].

The rate of landcover transition over this period is also changing. We estimate an 84% increase in the rate of expansion of graminoid-dominated sites (Fen) when comparing the 1970–2000 (0.023 ha yr^−1^) and 2000–2014 (0.043 ha yr^−1^) time periods. The decrease in Palsa areas is, however, slowing down with a loss of 11% of its area over the 45-year period and a decrease in the loss rate during the most recent time period (2000–2014). While Bog area increased from the 1970–2000 period, there was an overall loss of 11% of Bog area for the full 45-year period. The decrease in Palsa and Bog dominated sites and increase in Fen and Open Water landcover types are likely due to increases in soil moisture from thaw in areas of the mire that are more hydrologically active [40] which encourages the establishment of sedge species that emit higher rates of CH_4_ and are more productive [41]. These changes in landcover, as well as the rates of transition, are important parameters that should be incorporated to predictive models if we are able to adequately assess the feedbacks to climate from permafrost peatlands [42].

### (b) Stordalen Mire C balance

Permafrost peatlands have remained a C sink for many thousands of years [43,44], however, amplified Arctic warming could reverse this trend. Recent model predictions using global warming stabilization scenarios for northern peatlands project these ecosystems will turn from the current C sink to a temporary C source as permafrost-affected peatlands thaw (on the order of 25–75 years) [27]. The net carbon balance of the Stordalen Mire over the past 45 years has remained negative (C sink) even though CH_4_ emissions are increasing (figure 4). Our findings diverge from current permafrost peatland model predictions indicating that during active thermokarsting (or permafrost collapse as observed at Stordalen Mire), these systems are a net C source [27]. The inconsistencies between model predictions and field observations could be due to the increasing rates of transition of landcover from permafrost to higher productivity landcover types (Fen) [42] as well as local environmental variables that affect the presence or absence of permafrost: changes in snow cover, topography, hydrology and soil type [45] that can not be adequately accounted for in large scale simulations.

Even though Stordalen Mire remains a strong net C sink, storing 3× the amount of C as CO_2_ than it emits as CH_4_, as the landscape shifts to larger areas of open water and fen, net CH_4_ emissions are increasing (figure 4). Open water areas, in particular small lakes and ponds, are a large source of CH_4_ to the atmosphere [46,47]. Post-glacial lakes and small ponds at Stordalen Mire emit significant amounts of CH_4_ [22,23,35]. Including CH_4_ emissions from open water ponds into the NCB for Stordalen Mire resulted in a shift from a strong to a weak C sink [35]. Graminoid species characteristic of fen sites at Stordalen Mire are responsible for the northern peatlands CH_4_ source due to the ability of these plants to transport of CH_4_ through aerenchyma, effectively bypassing oxidation [48,49]. Increasing the area of fen landcover will increase CH_4_ emissions in northern peatlands and in permafrost peatlands especially [50–52].

### (c) Stordalen Mire is serving as a positive feedback to climate change

In 1970, the net radiative forcing of Stordalen Mire was negative (net cooling) but by 2000 it became strongly positive. The trend continued with higher positive net radiative forcing in 2014, doubling over this 15-year period. The rate of change in the radiative forcing between the first 30 years and the last 15 years increased by 85%. This shift from weakly negative to strongly positive was driven by the increasing areas of Open Water and Fen landcover classes. Open water areas formed through permafrost collapse or thermokarst are predicted to increase with climate warming [53]. This collapse can allow for open water ponds to form then potentially transition to sedge dominated sites increasing CH_4_ emissions from these landscapes [22]. In some areas, however, thermokarst can lead to drainage [54] which could reverse net radiative forcing by replacing the water body with drained, oxygenated peat likely becoming a CO_2_ source and reducing CH_4_ emissions [55].

Previous climate forcing estimates for the Stordalen Mire showed that this landscape had positive climate forcing during the growing seasons for the 1970 and 2000 time periods [32]. The discrepancy between these previous estimates and our study is partly due to our use of updated trace gas exchange rates that are based mostly on measurements taken all year long (table 1 and figure 3). Johansson *et al*. [32] reported the best available data that was based on growing season measurements likely exaggerating the impact of CH_4_ emissions. Additionally, our estimates are using the CH_4_ GWP_100 of 28 which has since been increased from 23 between IPCC AR4 and AR5 [24]. This change was due to new estimates of the atmospheric lifetimes, impulse response functions and radiative efficiencies for both CO_2_ and CH_4_, as well as the indirect effects factor used for CH_4_ [24,56]. Since all of these factors depend on atmospheric concentrations, ongoing increases in the atmospheric burdens of CO_2_ and CH_4_ can be expected to cause future changes in CH_4_ climate forcing impact and GWP values, likely increasing the radiative forcing of thawing permafrost landscapes like Stordalen Mire. A new, higher GWP_100 value for CH_4_, 32 ± 14%, has been published [57]; the IPCC AR5 value used here is at the lower end of that uncertainty range. Using this GWP_100 of 32 does not affect the trend in radiative forcing over time but does significantly increase the net radiative forcing of Stordalen Mire (in 2014 it goes from 5100 kg CO_2-equiv_ yr^−1^ to 9200 kg CO_2-equiv_ yr^−1^) highlighting the importance of differences in CH_4_ fluxes in the mire's net radiative forcing.

Radiative forcing of peatlands across the Holocene indicates that due to net C accumulation, they have had a cooling impact on the atmosphere for the last 11 000 years [25,26]. As permafrost peatlands thaw, this cooling trend could change at least until ecosystems equilibrate to a new steady state [27], however, this is likely to occur heterogeneously across Arctic landscapes [26]. At Stordalen Mire, relatively small changes in per cent cover of highly productive sedges had a large impact on the net radiative forcing. Schaefer *et al*. [50] estimated that CH_4_ emissions resulting from permafrost thaw will contribute to no more than 16% of the warming, while other estimates are more than double that [51]. Capturing the trend in radiative forcing and then predicting it into the future requires year-round measurement of trace gas exchange and monitoring of small scale landcover change that captures the impact of thawing on hydrology and vegetation community composition.

## 5. Conclusion

Using observations from 1970 to 2014, we have shown that Stordalen Mire, a thawing permafrost peatland in northern Sweden, has behaved as a carbon sink over this time period, providing a valuable ecosystem service, however, the net trace gas radiative balance has gone from slightly negative to highly positive over this 45-year period due to change in landcover from Palsa and Bog dominated areas to Fen and Open Water. These results indicate that changes in actively thawing permafrost ecosystems can be observed on decadal timescales and that these ecosystems are acting as a positive feedback to climate change. It is becoming ever more important to monitor this change in these climate-vulnerable regions in an effort to determine the state of these ecosystems and to understand the response as the Arctic continues to experience unprecedented warming.

## Data accessibility

All data used in this study are available at https://isogenie-db.asc.ohio-state.edu/.
